# Effects of HBsAg carriers on pregnancy complications in pregnant women: a retrospective cohort study

**DOI:** 10.3389/fmed.2023.1166530

**Published:** 2023-05-24

**Authors:** Mengqing Weng, Jie Wang, Jingfeng Yin, Wenning Ren, Caiping Wei, Wenshan Yang, Huimin He

**Affiliations:** ^1^School of Information and Management, Guangxi Medical University, Nanning, China; ^2^Department of Gynaecology, Longhua District People's Hospital, Shenzhen, China; ^3^Medical Records Library, Longhua District People's Hospital, Shenzhen, China; ^4^School of Life Sciences, Central South University, Changsha, China

**Keywords:** HBsAg carrier, pregnancy complications, pregnancy induced hypertension, intrahepatic cholestasis of pregnancy, hypothyroidism of pregnancy

## Abstract

**Objective:**

Hepatitis B virus (HBV) infection is a major health threat worldwide, especially in developing countries. We aimed to investigate the impact of hepatitis B carrier on pregnancy complications in pregnant women, in China.

**Methods:**

This retrospective cohort study was conducted by using data from the EHR system of Longhua District People’s Hospital in Shenzhen, China, from January 2018 to June 2022. Binary logistic regression was used to evaluate the relationship between HBsAg carrier status and pregnancy complications and pregnancy outcomes.

**Results:**

The study included 2095 HBsAg carriers (exposed group) and 23,019 normal pregnant women (unexposed group). Pregnant women in the exposed group were older than the pregnant women in the unexposed group (29 (27,32) vs. 29 (26,32), *p* < 0.001). In addition, the incidence of some adverse pregnancy complications in the exposure group was lower than that in the unexposed group, including hypothyroidism of pregnancy (adjusted odds ratio [aOR], 0.779; 95% confidence interval [CI], 0.617–0.984; *p* = 0.036), hyperthyroidism of pregnancy (aOR, 0.388; 95% CI, 0.159–0.984; *p* = 0.038), pregnancy induced hypertension (aOR, 0.699; 95% CI, 0.551–0.887; *p* = 0.003), antepartum hemorrhage (aOR, 0.294; 95% CI, 0.093–0.929; *p* = 0.037). However, compared with the unexposed group, the exposed group had a higher risk of lower birth weight (aOR, 1.12; 95% CI, 1.02–1.23; *p* = 0.018) and intrahepatic cholestasis of pregnancy (aOR, 2.888, 95% CI, 2.207–3.780; *p* < 0.001).

**Conclusion:**

The prevalence rate of HBsAg carriers in pregnant women in Longhua District of Shenzhen was 8.34%. Compared with normal pregnant women, HBsAg carriers have a higher risk of ICP, a lower risk of gestational hypothyroidism and PIH, and a lower birth weight of their infants.

## Introduction

Hepatitis B virus (HBV) infection is a major health threat worldwide. In 2019, about 296 million people worldwide were infected with HBV, and 1.5 million new infections occur each year. The geographic distribution of HBV infection is highly heterogeneous, with HBV prevalence levels above 7% (African Region) and less than 2% (Region of the Americas) (1). The largest burden of HBV infection is in China (2), where nearly 23,355,000 cases of HBV infection occurred in 2019 (about 29.0% of global HBV infections) (3), and the maternal infection rate was as high as 5.58% (4). In areas with high prevalence of HBV infection, although successfully introduced large-scale hepatitis b vaccination, vertical transmission is the main mode of infection, due to the high maternal viral load or HBV/DNA levels, maternal micro blood transfusion or mother blood leakage etc., (5), each year about 4 million to 5 million children around the world by their mothers infected with HBV (6). If maternal HBV infection affects pregnancy, it may also affect the survival of offspring. Therefore, the key question is, does maternal HBV infection affect pregnancy?

In this field, there are few reports on the association between HBsAg carriers and pregnancy complications and the relationship between them is unclear. Chen et al. reported that HBsAg-positive pregnant women have an increasing risk of intrahepatic cholestasis of pregnancy (ICP), low birth weight, fallopian cysts, cesarean scar pregnancy, thrombocytopenia, and fetal distress (7). Wan et al. found that maternal HBsAg carrying was associated with increasing risk of pregnancy-induced hypertension, fetal distress, cesarean delivery and macrosomia (8). However, Sun et al. found that the incidence of labor induction and small-for-gestational age (SGA) infants in HBV carriers was lower than that in the normal group, and there were no significant differences in pregnancy complications, delivery or perinatal outcomes (9). Huang et al. found that maternal chronic HBV infection will occur without increasing the risk of pregnancy-induced hypertension (10).

Obviously, the current research on the relationship between hepatitis B carriers and pregnancy complications and outcomes is insufficient. Therefore, this study aims to explore the influence of HBsAg carrier status during pregnancy on pregnancy complications and outcomes in the population of Longhua District, Shenzhen, China (Its population is 2.535 million, more than most cities in China). This is a retrospective cohort study to analyze the relationship between pregnancy complications and outcomes in 23,019 normal pregnant women and 2095 pregnant women were the HBsAg carrier.

## Materials and methods

### Subject

**Figure 1:**
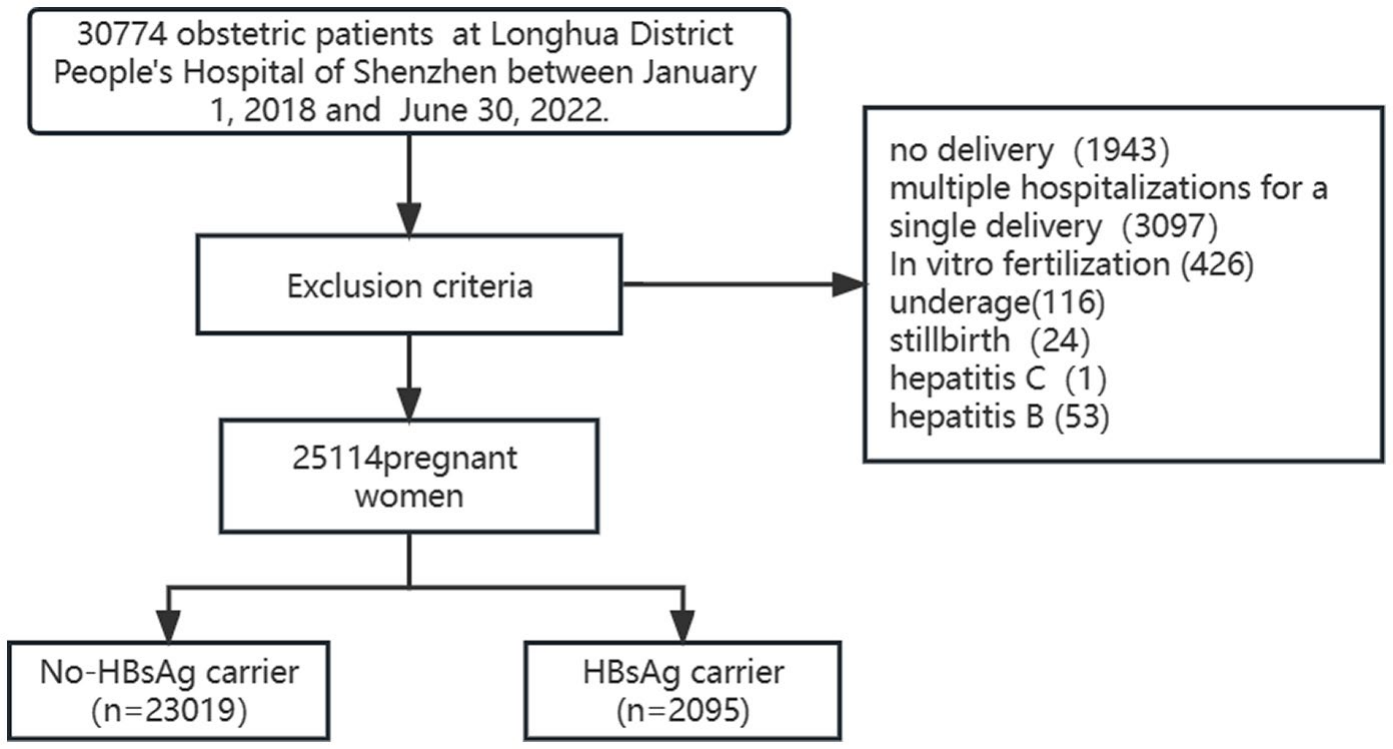
Flowchat for this study. Data exclusion criteria.

This was a retrospective study based on data from the Electronic Medical Record (EMR) information system for the period January 2018 to June 2022 in Longhua District People’s Hospital of Shenzhen, a tertiary referral hospital located in China Special Economic Zone. Only anonymized data from the EMR information system were used in this study. No patients were included as study participants. After filtering by exclusion criteria, 23,019 normal pregnant women (unexposed group) and 2095 HBsAg carrier pregnant women (exposed group) were included in this study (Figure 1). HBsAg carriers were identified by obstetricians based on the criteria of serological.

### Inclusion and exclusion criteria

All pregnant women in Shenzhen were tested for the hepatitis B triad at the first prenatal visit (11–13 weeks of gestation), and HBV-DNA was tested for HBHBS-positive patients. Patients were excluded as follows: (1) patient was a single pregnancy but hospitalized multiple times (2) patient was hospitalized for purposes other than delivery (3) stillbirth (4) underage (5) *in vitro* fertilization (Ivf) (6) chronic viral hepatitis B infection (7) chronic viral hepatitis C infection (8) incomplete data. This study was authorized by our hospital ethics committee. (Longhua District People’s Hospital Ethical Review (Research) [2023] No. (001)).

### Outcome measurements

Complications of pregnancy include pregnancy induced hypertension (PIH), gestational diabetes mellitus (GDM), intrahepatic cholestasis of pregnancy (ICP), anemia of pregnancy, hypothyroidism of pregnancy, hyperthyroidism of pregnancy, and digestive diseases of pregnancy. Pregnancy outcomes include premature rupture of fetal membranes, meconium-staining amniotic fluid, premature delivery, Neonatal weight, postpartum hemorrhage, and cesarean section.

### Statistical analysis

The statistical package SPSS Statistics 24.0 (IBM-SPSS, Chicago, United States) was used for data analysis. Quantitative data was expressed as mean ± standard deviation (SD) or quartile and qualitative data as percentage (%). For univariate analysis, the t-test was used when the quantitative data obeyed normal distribution, and the Mann–Whitney u test was used when the data did not obey normal distribution; Pearson *X*^2^ test or Fisher test were used for qualitative data. Binary Logistic regression analysis was used to detect the relationship between HBsAg carrier and pregnancy outcomes and complications. *p* < 0.05 was considered statistically significant. Results were expressed in terms of odds ratios (OR) and adjusted odds ratios (aOR), with their respective 95% confidence intervals (95%CI). Variables in step 1 were neonatal weight, hypothyroidism of pregnancy, hyperthyroidism of pregnancy, PIH, ICP, antepartum hemorrhage, ethnicity, maternal age. In step 2, adjusted for maternal age and ethnicity, variables.

## Results

### Basic demographic data

Among the 25,114 pregnant women in the database, 2095 (8.34%) HBsAg carriers were identified. Less than the Guangdong HBV prevalence (9.84%) in the study of *Jue Liu et al* (4). The average age of the HBsAg carrier group was 29 (27, 32) years, which was higher than that of the unexposed group 29 (26, 32) years (*Z* = −3.033, *p* = 0.002). The ethnic minority proportion in the HBsAg carrier group was 2.48% (52), significantly less than 5.18% (1192) in the unexposed group (*X*^2^ = 29.650, *p* < 0.001). The mean length of hospital stay between the HBsAg carrier group and the unexposed groups was not statistically significant (*Z* = −1.694, *p* = 0.090). As shown in Table 1.

**Table 1:** Univariate analysis of demographic and clinical characteristics of cohort.

Variables	No HBsAg carrier (23019)	HBsAg carrier (2095)	*z*/*x*^2^	*p*
Maternal age (years)	29 (26,32)	29 (27,32)	−3.033	0.002^a^
Maternal age (%)			16.611	<0.001^b^
<25 years	2,859 (12.42)	202 (9.64)		
25-35 years	17,618 (76.54)	1,679 (80.14)		
>35 years	2,542 (11.04)	214 (10.21)		
Ethnicity (Minority) (%)	1,192 (5.18)	52 (2.48)	29.650	<0.001^b^
Hospital day (days)	4 (3.5)	4 (3.5)	−1.694	0.090
Pregnancy induced hypertension (%)	1,132 (4.92)	76 (3.63)	6.979	0.008^a^
Gestational diabetes mellitus (%)	2,942 (12.78)	281 (13.41)	0.686	0.408
Intrahepatic cholestasis of pregnancy (%)	271 (1.18)	69 (3.29)	64.395	<0.001^b^
Anemia of pregnancy (%)	14,247 (61.89)	1,315 (62.77)	0.625	0.429
Hypothyroidism of pregnancy (%)	1,093 (4.75)	79 (3.77)	4.123	0.042^a^
Hyperthyroidism of pregnancy (%)	137 (0.60)	5 (0.24)	4.341	0.037^a^
Digestive system diseases of pregnancy (%)	119 (0.52)	15 (0.72)	1.433	0.231
Antepartum hemorrhage (%)	103 (0.45)	3 (0.14)	4.229	0.040^a^
Pelvic adhesion (%)	3,184 (13.83)	293 (13.99)	0.038	0.845
Thrombocytopenia (%)	73 (0.32)	7 (0.33)	0.017	0.895
Oligohydramnios (%)	647 (2.81)	56 (2.67)	0.134	0.715

^a^ represents *p* < 0.05; ^b^ represents *p* < 0.001. *p* < 0.05 were considered significant.

### Univariate analysis of influencing factors

Among the pregnancy complications, PIH, hypothyroidism of pregnancy, hyperthyroidism of pregnancy, antepartum hemorrhage and ICP showed statistically significant differences between HBsAg carrier group and non-exposed group (all *p* < 0.05). In pregnancy outcomes, only the difference in neonatal birth weight was statistically significant between the two groups (*p* = 0.018). The incidence of gestational diabetes mellitus, anemia of pregnancy, pelvic adhesion, thrombocytopenia, oligohydramnios, premature birth, postpartum hemorrhage, cesarean section, obstructed labor, meconium-staining amniotic fluid and premature rupture of fetal membranes did not differ significantly between the HBsAg carrier group and non-exposed groups (all *p* > 0.05). As shown in Tables 1, 2.

**Table 2:** Univariate analysis of pregnancy outcomes with HBsAg-positve and negative group.

Variables	No HBsAg carrier (23019)	HBsAg carrier (2095)	z/*X*^2^	*p*
Neonatal weight (Kg)	3.25 (3.00,3.50)	3.20 (2.95,3.50)	−2.376	0.018^a^
Premature birth (%)	1,254 (5.45)	124 (5.92)	0.822	0.365
Postpartum hemorrhage (%)	355 (1.54)	33 (1.58)	0.014	0.907
Cesarean section (%)	7,126 (30.96)	643 (30.69)	0.063	0.802
Obstructed labor (%)	384 (1.67)	36 (1.72)	0.029	0.864
Meconium-staining amniotic fluid (%)	118 (0.51)	8 (0.38)	0.658	0.417
Premature rupture of fetal membranes	5,260 (22.85)	449 (21.43)	2.200	0.138

^a^ represents *p* < 0.05. *p* < 0.05 were considered significant.

### Analysis of influencing factors

The results of the multivariate analysis were slightly altered when adjusted for maternal age and ethnicity. Compared with the unexposed group, the exposed group had a higher risk of lower birth weight (aOR, 1.12; 95% CI, 1.02–1.23; *p* = 0.018) and intrahepatic cholestasis of pregnancy (aOR, 2.888, 95% CI, 2.207–3.780; *p* < 0.001) The HBsAg carrier group had lower rates of some adverse pregnancy complications, including hypothyroidism of pregnancy (aOR, 0.779; 95% CI, 0.617–0.984; *p* = 0.036), hyperthyroidism of pregnancy (aOR, 0.388; 95% CI, 0.159–0.984; *p* = 0.038), PIH (aOR, 0.699; 95% CI,0.551–0.887; *p* = 0.003), antepartum hemorrhage (aOR, 0.294; 95% CI, 0.093–0.929; *p* = 0.037). As shown in Table 3.

**Table 3:** Multifactorial analysis of maternal HBsAg carrier in relation to pregnancy complications.

Variables	*p*	OR	95% CI for OR	*p*	aOR	95%CI for aOR
Neonatal weight	0.009^a^	0.880	0.799–0.968	0.018^a^	0.891	0.810–0.981
Hypothyroidism of pregnancy	0.033^a^	0.776	0.614–0.980	0.036^a^	0.779	0.617–0.984
Hyperthyroidism of pregnancy	0.040^a^	0.391	0.160–0.958	0.038^a^	0.388	0.159–0.948
Pregnancy induced hypertension	0.004^a^	0.707	0.557–0.898	0.003^a^	0.699	0.551–0.887
Antepartum hemorrhage	0.037^a^	0.293	0.093–0.929	0.037^a^	0.294	0.093–0.929
Intrahepatic cholestasis of pregnancy	<0.001^b^	2.901	2.215–3.799	<0.001^b^	2.888	2.207–3.780
Ethnicity	<0.001^b^	2.121	1.600–2.812	–	–	–
Maternal age (years)						
<25 year	<0.001^b^	0.730	0.627–0.850	–	–	–
>35 year	0.189	0.905	0.780–1.050	–	–	–
Constant	<0.001^b^	0.071	–	–	–	–

The reference category for ethnicity is minority; the reference category for maternal age is 25–35 years.

OR, odds ratio; aOR, adjusted odds ratio; CI, confidence intervals.

^a^ represents *p* < 0.05; ^b^ represents *p* < 0.001. *p* < 0.05 were considered significant.

## Discussion

The percentage of HBsAg carriers among pregnant women in this study was 8.34%, higher than the average infection rate in China (5.44%), but slightly lower than the average infection rate of Guangdong Province (9.84%) (4). One of the reasons may be that this study did not include women who had early abortion, infertility, and did not receive services from our hospital, so the data collection was not comprehensive enough. Another reason may be that Shenzhen is one of the largest cities in China and attracts many people from various provinces, so it leads to a slightly lower infection rate than the overall rate in Guangdong Province. Basically, consistent with previous reports in the relevant literature (11, 12), the maternal age of HBsAg carriers was slightly higher than that in the non-exposed group. The prevalence of infection in the <25 years age group was about 6.6%, slightly lower than in the 25–35 years and > 35 years age groups, which should be related to the introduction of hepatitis B vaccination in China since 1992 (13).

We found that pregnant women with HBsAg carriers had a higher risk of ICP compared with the non-exposed group (aOR = 2.888). This is consistent with past studies that HBsAg carrier is a high risk factor for the development of ICP, but the results of this study showed that the risk of ICP in pregnant women with HBsAg carriage was 2.888 times higher than that of normal mothers, which is higher than previous studies (14–16). Jiang et al. Meta-analyses showed that not only were pregnant women infected with HBV at increased risk of developing ICP, but also patients with ICP were at increased risk of HBV infection (17). Therefore, in clinical practice, we should pay more attention to the serum total bile acid level and clinical manifestations of HBsAg carriers, and do a good job in the early prevention, diagnosis and treatment of ICP.

Pregnant women with HBsAg carriers have a lower risk of PIH (aOR = 0.699). Zhang et al. found that maternal HBsAg carrier group had a reduced risk of pre-eclampsia, premature rupture of membranes and PIH (OR = 0.83) (18). *William* et al. also found the risk of infecting PIH in HBsAg positive mothers (OR = 0.55) is lower than the in HBsAg negative mothers (19). However, in a case–control study, HBV-infected women reported a higher risk of PIH than the normal group (OR = 4.2) (20). The bias in these results may be due to differences in research methods and the cohort used in the study. Although the mechanism of the relationship between maternal HBsAg carrier and the incidence of PIH is unknown, PIH may be associated with an altered maternal immune response to fetal allograft, resulting in increased immune tolerance and decreased incidence of PIH.

Pregnant women with HBsAg carrier had a lower risk of developing hypothyroidism in pregnancy (aOR = 0.779). In contrast, no effect of hepatitis B virus status on thyroid status was found in the study by Testa and Percy et al. (21, 22). But instead of pregnant women, they studied people with autoimmune thyroid disease and the elderly, so the effect of hepatitis B virus on thyroid function may vary between study subjects. The liver is the target organ of thyroid hormone and it also plays an important role in hormone metabolism (23). In the study of Wu and Feng et al. showed that liver disorders were negatively correlated with TSH (24) and TT4 and FT4 were positively correlated (25). In turn, FSH, TT4, FT4 are important indicators for the diagnosis of hypothyroidism, so HBsAg carrier has a protective effect against hypothyroidism in pregnancy, which is worthy of clinical attention.

Birth weight of newborns in the HBsAg carrier group was lower than in the unexposed group (OR = 1.14). *Unal* et al. also found that HBV infection could lead to the decrease of newborn weight (26). Secondly, a retrospective cohort study in Thailand also found that HBsAg-positive women had a higher risk of having low birth weight babies (RR = 1.258) (27). Taken together, these studies suggest that HBsAg carriers are a high risk factor for neonatal weight, and clinical monitoring of neonatal weight in HBsAg carriers should be strengthened.

Some studies have reported that there is no association between GDM and HBV infection. For example, *Cui* et al. showed that there was no difference between the probability of HBV carriers suffering from GDM and the non-exposed group (28). The research results of Chen et al. also confirmed this point (7). However, some studies have shown an association between maternal HBV infection and GDM. For example, Giles et al.’s study showed that women with HBV infection had an increasing risk of developing GDM (OR = 1.2) (29). Consistenting with the former, we found that maternal HBsAg carriers were not associated with the risk of GDM infection.

Pregnant women with HBsAg carrier have a lower risk of antepartum hemorrhage and (aOR = 0.294) hyperthyroidism in pregnancy (aOR = 0.388). The liver is necessary for coagulation because it is the organ that produces prothrombin and other coagulation factors, and the liver is the target organ for thyroid hormones. HBsAg carrier was found to be a protective factor for prenatal hemorrhage and hyperthyroidism in pregnancy in this study, which is different from (30–32). However, since the sample size of both in this study was less than 1%, there are some limitations in the results and more studies are needed to prove it if it needs to be practiced in the clinic.

The study also found no association between preterm delivery, premature rupture of membranes, cesarean section, meconium-stained amniotic fluidis and HBV infection, consistent with studies (7, 9, 33, 34). Interestingly, it has been shown in some studies (35–37) that maternal HBsAg carriers may have an increased risk of preterm birth. And in an Israeli study it was noted that HBV/HCV infected pregnant women have higher rates of premature rupture of membranes, placental abruption, labor induction, cesarean section, rates of perinatal mortality, and congenital malformations (38). The reasons for the inconsistent results of the studies are unclear and may be due, on the one hand, to the fact that the studies were based on different sites and different populations and, secondly, may be related to the viral load of pregnant women with HBsAg carriers.

We studied the relationship between maternal HBsAg carriers and pregnancy complications among pregnant women in Longhua District, Shenzhen City. Although the sample size of our study is relatively large, there are still some limitations. First, although our sample size is large, this sample size only represents the Longhua District of Shenzhen. Even though the resident population of Longhua District in Shenzhen has exceeded 2.5 million, which is well above the level of a large city. Second, the study did not include potential confounding factors that could affect pregnancy outcome. For example, risk factors such as smoking and alcohol consumption should be taken into consideration. In addition, our data on viral load in HBV carriers is poorly documented, so whether viral load affects adverse pregnancy complications and outcomes is uncertain.

In conclusion, the prevalence rate of HBsAg carriers in pregnant women in Longhua District, Shenzhen City, China was 8.34%. Compared with normal pregnant women, HBsAg carriers have a higher risk of ICP, a lower risk of gestational hypothyroidism and PIH, and a lower birth weight of their infants. Maternal HBsAg carriers were not associated with the occurrence of these adverse pregnancy outcomes or complications, including GDM, anemia of pregnancy, pelvic adhesion, thrombocytopenia, oligohydramnios, premature birth, postpartum hemorrhage, cesarean section, obstructed labor, meconium-staining amniotic fluid and premature rupture of fetal membranes.

## Data availability statement

The datasets presented in this article are not readily available because Data cannot be shared publicly because of restrictions apply to the availability of these data, which were used under license for the current study. Data are available from corresponding author for researchers who meet the criteria for access to confidential data. Requests to access the datasets should be directed to HH, 2422190004@qq.com.

## Ethics statement

The studies involving human participants were reviewed and approved by Medical Ethics Committee of Shenzhen Longhua District People's Hospital. Written informed consent for participation was not required for this study in accordance with the national legislation and the institutional requirements. No potentially identifiable images or data are included in this article.

## Author contributions

MW: methodology, data analysis, and writing–original draft. JW: conceptualization, resources, and writing–review. JY: methodology and writing–review. WR and CW: data analysis and writing–review. WY: conceptualization and writing–review. HH: resources, writing–review, and supervision. All authors contributed to the article and approved the submitted version.

## Funding

This research was funded by Department of Higher Education, Ministry of Education of China, 2021 Second Batch of Industry-University Cooperative Education Project (202102490073).

## Conflict of interest

The authors declare that the research was conducted in the absence of any commercial or financial relationships that could be construed as a potential conflict of interest.

## Publisher’s note

All claims expressed in this article are solely those of the authors and do not necessarily represent those of their affiliated organizations, or those of the publisher, the editors and the reviewers. Any product that may be evaluated in this article, or claim that may be made by its manufacturer, is not guaranteed or endorsed by the publisher.

## Abbreviations

HBV: Hepatitis B virus
HBsAg: Hepatitis B surface antigen
GDM: Gestational diabetes mellitus
PIH: pregnancy induced hypertension
OR: odds ratio
aOR: adjusted odds ratio
CI: confidence intervals
ICP: intrahepatic cholestasis of pregnancy
Ivf: If the patient is *in vitro* fertilization
SD: Standard deviation

## References

[1] Organization, W.H, Global progress report on HIV, viral hepatitis and sexually transmitted infections, (2021). Available at: https://www.who.int/publications/i/item/9789240027077 (Accessed July 15, 2021).

[2] Liu J Liang W Jing W Liu M. Countdown to 2030: eliminating hepatitis B disease. China Bull World Health Organ. (2019) 97:230–8. doi: 10.2471/blt.18.219469, PMID: 30992636 PMC6453311

[3] Yue T Zhang Q Cai T Xu M Zhu H Pourkarim MR. Trends in the disease burden of HBV and HCV infection in China from 1990-2019. Int J Infect Dis. (2022) 122:476–85. doi: 10.1016/j.ijid.2022.06.017, PMID: 35724827

[4] Liu J Wang X Wang Q Qiao Y Jin X Li Z. Hepatitis B virus infection among 90 million pregnant women in 2853 Chinese counties, 2015-2020: a national observational study. Lancet Reg Health West Pac. (2021) 16:100267. doi: 10.1016/j.lanwpc.2021.100267, PMID: 34590067 PMC8429967

[5] Pawlowska M Pniewska A Pilarczyk M Kozielewicz D Domagalski K. Prophylaxis of vertical HBV infection. Expert Opin Drug Saf. (2016) 15:1361–8. doi: 10.1080/14740338.2016.1211106 27402246

[6] Thio CL Guo N Xie C Nelson KE Ehrhardt S. Global elimination of mother-to-child transmission of hepatitis B: revisiting the current strategy. Lancet Infect Dis. (2015) 15:981–5. doi: 10.1016/s1473-3099(15)00158-9 26145195

[7] Chen YM Ning WW Wang X Chen YJ Wu B Tao J. Maternal hepatitis B surface antigen carrier status and pregnancy outcome: a retrospective cohort study. Epidemiol Infect. (2022) 150:1–22. doi: 10.1017/s0950268822000681, PMID: 35440355 PMC9102056

[8] Wan ZH Zhou AF Zhu HP Lin XF Hu D Peng SX. Maternal hepatitis B virus infection and pregnancy outcomes: a hospital-based case-control study in Wuhan. China J Clin Gastroenterol. (2018) 52:73–8. doi: 10.1097/mcg.0000000000000842 28723858

[9] Sun Q Lao TT Du MY Xie M Sun YH Bai B. Chronic maternal hepatitis B virus infection and pregnancy outcome-a single center study in Kunming, China. BMC Infect Dis. (2021) 21:21. doi: 10.1186/s12879-021-05946-7, PMID: 33691634 PMC7945294

[10] Huang X Tan H Li X Zhou S Wen SW Luo M. Maternal chronic HBV infection would not increase the risk of pregnancy-induced hypertension –results from pregnancy cohort in Liuyang rural China. PLoS One. (2014) 9:e114248. doi: 10.1371/journal.pone.0114248, PMID: 25479003 PMC4257699

[11] Zhao Y Chen Y-L Song H-Q Huang P-Y Wang L-Y Liu W. Effects of maternal hepatitis B surface antigen positive status on the pregnancy outcomes: a retrospective study in Xiamen, China, 2011-2018. PLoS One. (2020) 15:e0229732. doi: 10.1371/journal.pone.0229732, PMID: 32155166 PMC7064202

[12] Katamba PS Mukunya D Kwesiga D Nankabirwa V. Prenatal hepatitis B screening and associated factors in a high prevalence district of lira, northern Uganda: a community based cross sectional study. BMC Public Health. (2019) 19:1004. doi: 10.1186/s12889-019-7344-6, PMID: 31349838 PMC6660940

[13] Luo ZB Li LJ Ruan B. Impact of the implementation of a vaccination strategy on hepatitis B virus infections in China over a 20-year period. Int J Infect Dis. (2012) 16:E82–8. doi: 10.1016/j.ijid.2011.10.009, PMID: 22178658

[14] Tan J Liu X Mao X Yu J Chen M Li Y. HBsAg positivity during pregnancy and adverse maternal outcomes: a retrospective cohort analysis. J Viral Hepat. (2016) 23:812–9. doi: 10.1111/jvh.12545, PMID: 27167604

[15] Cai Q Liu H Han W Liu L Xu Y He Y. Maternal HBsAg carriers and adverse pregnancy outcomes: a hospital-based prospective cohort analysis. J Viral Hepat. (2019) 26:1011–8. doi: 10.1111/jvh.13105, PMID: 30972911

[16] Arthuis C Diguisto C Lorphelin H Dochez V Simon E Perrotin F. Perinatal outcomes of intrahepatic cholestasis during pregnancy: an 8-year case-control study. PLoS One. (2020) 15:e0228213. doi: 10.1371/journal.pone.0228213, PMID: 32074108 PMC7029845

[17] Jiang R Wang T Yao Y Zhou F Huang X. Hepatitis B infection and intrahepatic cholestasis of pregnancy: a systematic review and meta-analysis. Medicine. (2020) 99:e21416. doi: 10.1097/MD.0000000000021416, PMID: 32756142 PMC7402766

[18] Zhang YL Chen JC Liao TT Chen SW Yan JY Lin XQ. Maternal HBsAg carriers and pregnancy outcomes: a retrospective cohort analysis of 85,190 pregnancies. BMC Pregnancy Childbirth. (2020) 20:724. doi: 10.1186/s12884-020-03257-4, PMID: 33238912 PMC7687687

[19] To, W.W.K Cheung W Mok K-M. Hepatitis B surface antigen carrier status and its correlation to gestational hypertension. Aust N Z J Obstet Gynaecol. (2003) 43:119–22. doi: 10.1046/j.0004-8666.2003.00029.x, PMID: 14712966

[20] Saleh-Gargari S Hantoushzadeh S Zendehdel N Jamal A Aghdam H. The Association of Maternal HBsAg carrier status and perinatal outcome. Hepat Mon. (2009) 9:180–4.

[21] Testa A Castaldi P Fant V Fiore GF Grieco V De Rosa A. Prevalence of HCV antibodies in autoimmune thyroid disease. Eur Rev Med Pharmacol Sci. (2006) 10:183–6. PMID: 16910348

[22] Percy ME Potyomkina Z Dalton AJ Fedor B Mehta P Andrews DF. Relation between apolipoprotein E genotype, hepatitis b virus status, and thyroid status in a sample of older persons with down syndrome. Am J Med Genet A. (2003) 120A:191–8. doi: 10.1002/ajmg.a.20099, PMID: 12833399

[23] Malik R Hodgson H. The relationship between the thyroid gland and the liver. QJM: Int J Med. (2002) 95:559–69. doi: 10.1093/qjmed/95.9.559 12205333

[24] Wu YC You SL Zang H Liu HT Mao YL Mao PY. Usefulness of serum thyroid-stimulation hormone (TSH) as a prognostic indicator for acute-on-chronic liver failure. Ann Hepatol. (2015) 14:218–24. doi: 10.1016/s1665-2681(19)30784-7, PMID: 25671831

[25] Feng H-L Li Q Cao W-K Yang J-M. Changes in thyroid function in patients with liver failure and their clinical significance: a clinical study of non-thyroidal illness syndrome in patients with liver failure. Hepatobiliary Pancreat Dis Int. (2020) 19:561–6. doi: 10.1016/j.hbpd.2020.05.001, PMID: 32535064

[26] Unal C Tanacan A Ziyadova G Fadiloglu E Beksac MS. Effect of viral load on pregnancy outcomes in chronic hepatitis B infection. J Obstet Gynaecol Res. (2019) 45:1837–42. doi: 10.1111/jog.14065 31332897

[27] Sirilert S Traisrisilp K Sirivatanapa P Tongsong T. Pregnancy outcomes among chronic carriers of hepatitis B virus. Int J Gynecol Obstet. (2014) 126:106–10. doi: 10.1016/j.ijgo.2014.02.019, PMID: 24834849

[28] Cui AM Cheng XY Shao JG Li HB Wang XL Shen Y. Maternal hepatitis B virus carrier status and pregnancy outcomes: a prospective cohort study. BMC Pregnancy Childbirth. (2016) 16:87. doi: 10.1186/s12884-016-0884-1, PMID: 27113723 PMC4845477

[29] Giles M Davey M-A Wallace E. Chronic hepatitis B infection and the risk of gestational diabetes: a cross-sectional study. BJOG Int J Obstet Gynaecol. (2020) 127:1147–52. doi: 10.1111/1471-0528.16217, PMID: 32176400

[30] Reddick KLB Jhaveri R Gandhi M James AH Swamy GK. Pregnancy outcomes associated with viral hepatitis. J Viral Hepat. (2011) 18:e394–8. doi: 10.1111/j.1365-2893.2011.01436.x 21692952

[31] Chen B Wang Y Lange M Kushner T. Hepatitis C is associated with more adverse pregnancy outcomes than hepatitis B: a 7-year national inpatient sample study. Hepatol Commun. (2022) 6:2465–73. doi: 10.1002/hep4.2002, PMID: 35748104 PMC9426407

[32] Cui W Deng B Wang W Liu P. Graves’ hyperthyroidism accompanied with acute hepatitis B virus infection: an extrahepatic manifestation? Virol J. (2016) 13:80. doi: 10.1186/s12985-016-0537-z, PMID: 27206523 PMC4874021

[33] Huang Q-T Wei S-S Zhong M Hang L-L Xu Y-Y Cai G-X. Chronic hepatitis B infection and risk of preterm labor: a meta-analysis of observational studies. J Clin Virol. (2014) 61:3–8. doi: 10.1016/j.jcv.2014.06.006, PMID: 24973811

[34] Wong S Chan LY Yu V Ho L. Hepatitis B carrier and perinatal outcome in singleton pregnancy. Am J Perinatol. (1999) 16:0485–8. doi: 10.1055/s-1999-6802, PMID: 10774765

[35] Ma X Sun D Li C Ying J Yan Y. Chronic hepatitis B virus infection and preterm labor(birth) in pregnant women—an updated systematic review and meta-analysis. J Med Virol. (2018) 90:93–100. doi: 10.1055/s-1999-6802, PMID: 28851115

[36] Peng S Chen H Li X Du Y Gan Y. Maternal age and educational level modify the association between chronic hepatitis B infection and preterm labor. BMC Pregnancy Childbirth. (2020) 20:38. doi: 10.1186/s12884-020-2729-1, PMID: 31937269 PMC6961340

[37] Xu C Bao Y Zuo J Li Y Tang Y Qu X. Maternal chronic hepatitis B virus infection and the risk of preterm birth: a retrospective cohort analysis in Chinese women. J Viral Hepat. (2021) 28:1422–30. doi: 10.1111/jvh.13585, PMID: 34342096

[38] Safir A Levy A Sikuler E Sheiner E. Maternal hepatitis B virus or hepatitis C virus carrier status as an independent risk factor for adverse perinatal outcome. Liver Int. (2010) 30:765–70. doi: 10.1111/j.1478-3231.2010.02218.x 20214739

